# Artificial Intelligence–Based Multimodal Risk Assessment Model for Surgical Site Infection (AMRAMS): Development and Validation Study

**DOI:** 10.2196/18186

**Published:** 2020-06-15

**Authors:** Weijia Chen, Zhijun Lu, Lijue You, Lingling Zhou, Jie Xu, Ken Chen

**Affiliations:** 1 Department of Anesthesiology Rui Jin Hospital, Luwan Branch Shanghai Jiao Tong University School of Medicine Shanghai China; 2 Department of Informatics Rui Jin Hospital, Luwan Branch Shanghai Jiao Tong University School of Medicine Shanghai China; 3 Department of Infection Prevention and Control Rui Jin Hospital, Luwan Branch Shanghai Jiao Tong University School of Medicine Shanghai China; 4 VitalStrategic Research Institute Shanghai China; 5 Synyi Research Shanghai China; 6 Precision Diagnosis and Image Guided Therapy Philips Research China Shanghai China

**Keywords:** surgical site infection, machine learning, deep learning, natural language processing, artificial intelligence, risk assessment model, routinely collected data, electronic medical record, neural network, word embedding

## Abstract

**Background:**

Surgical site infection (SSI) is one of the most common types of health care–associated infections. It increases mortality, prolongs hospital length of stay, and raises health care costs. Many institutions developed risk assessment models for SSI to help surgeons preoperatively identify high-risk patients and guide clinical intervention. However, most of these models had low accuracies.

**Objective:**

We aimed to provide a solution in the form of an Artificial intelligence–based Multimodal Risk Assessment Model for Surgical site infection (AMRAMS) for inpatients undergoing operations, using routinely collected clinical data. We internally and externally validated the discriminations of the models, which combined various machine learning and natural language processing techniques, and compared them with the National Nosocomial Infections Surveillance (NNIS) risk index.

**Methods:**

We retrieved inpatient records between January 1, 2014, and June 30, 2019, from the electronic medical record (EMR) system of Rui Jin Hospital, Luwan Branch, Shanghai, China. We used data from before July 1, 2018, as the development set for internal validation and the remaining data as the test set for external validation. We included patient demographics, preoperative lab results, and free-text preoperative notes as our features. We used word-embedding techniques to encode text information, and we trained the LASSO (least absolute shrinkage and selection operator) model, random forest model, gradient boosting decision tree (GBDT) model, convolutional neural network (CNN) model, and self-attention network model using the combined data. Surgeons manually scored the NNIS risk index values.

**Results:**

For internal bootstrapping validation, CNN yielded the highest mean area under the receiver operating characteristic curve (AUROC) of 0.889 (95% CI 0.886-0.892), and the paired-sample *t* test revealed statistically significant advantages as compared with other models (*P*<.001). The self-attention network yielded the second-highest mean AUROC of 0.882 (95% CI 0.878-0.886), but the AUROC was only numerically higher than the AUROC of the third-best model, GBDT with text embeddings (mean AUROC 0.881, 95% CI 0.878-0.884, *P*=.47). The AUROCs of LASSO, random forest, and GBDT models using text embeddings were statistically higher than the AUROCs of models not using text embeddings (*P*<.001). For external validation, the self-attention network yielded the highest AUROC of 0.879. CNN was the second-best model (AUROC 0.878), and GBDT with text embeddings was the third-best model (AUROC 0.872). The NNIS risk index scored by surgeons had an AUROC of 0.651.

**Conclusions:**

Our AMRAMS based on EMR data and deep learning methods—CNN and self-attention network—had significant advantages in terms of accuracy compared with other conventional machine learning methods and the NNIS risk index. Moreover, the semantic embeddings of preoperative notes improved the model performance further. Our models could replace the NNIS risk index to provide personalized guidance for the preoperative intervention of SSIs. Through this case, we offered an easy-to-implement solution for building multimodal RAMs for other similar scenarios.

## Introduction

Health care–associated infection (HAI) is a global patient safety problem, with surgical site infection (SSI) being one of the most common types of HAI [1-4]. The incidences of SSI for inpatients undergoing operations are 2%-5% in the United States [5], 2%-10% in Europe [6-9], and 4%-6% in China [10-13]. SSIs increase mortality and long-term disabilities, prolong hospital length of stay (LOS), and raise health care costs [1,5,11]. In China, SSIs prolong hospital LOS by 6-23 days and increase medical costs by US $2000-$6000 per patient, with the additional cost for one SSI patient needing to be offset by the medical revenue from 13 surgical patients [11].

In 2016, the World Health Organization recommended a large perioperative care bundle of interventions for preventing SSIs, which includes perioperative oxygen inhalation; maintenance of normal body temperature; maintenance of adequate glucose and circulating volume; use of sterile drapes, surgical gowns, wound-protector devices, and antimicrobial-coated sutures; provision of incisional wound irrigation; and prophylactic negative-pressure wound therapy [14]. However, the quality of evidence for most of these recommended interventions remains low. When we do not know whether these interventions are effective enough, using several interventions together is reasonable, and may even have a summation effect, for reducing the risk of SSI as much as possible. However, the shortcomings of bundle interventions are also apparent: they will consume large amounts of medical resources, especially when we strictly implement the recommendations of the guideline. Thus, data-driven guidance for personalized intervention is key to creating more effective SSI prevention and control programs.

Many institutions have developed risk assessment models (RAMs) focusing on SSIs to help surgeons preoperatively identify high-risk patients and guide clinical interventions. The most widely used traditional RAM is the National Nosocomial Infections Surveillance (NNIS) risk index [15], which is a scoring system ranging from 0 to 3. An American Society of Anesthesiologists (ASA) preoperative assessment score higher than 2; contaminated, dirty, or infected operation; and prolonged operation duration each account for 1 point in the NNIS risk index scoring system. The risk of SSI increases from 1.5% to 13.0% as the score goes up. Obviously, the three variables are easy to calculate, but are not enough to describe the characteristics of high-risk patients. To remedy these deficiencies, Mu et al included more patient- and hospital-specific variables and developed improved RAMs for each procedure under the 39 National Healthcare Safety Network (NHSN) procedure categories using stepwise logistic regression [16]. They trained these procedure-specific models using 849,659 patient records, from 2006 to 2008, from the NHSN database. Each model used 12-15 variables, including patient demographics, anesthesia, surgery, hospital settings, and NNIS risk index factors. The overall area under the receiver operating characteristic curve (AUROC) of the model reached 0.67, higher than the AUROC of the NNIS risk index, which is 0.60. The biggest problem with their RAM is that 39 different models need to be deployed together to achieve full functionality, which is cumbersome for clinical use. In a later study, Grant et al developed another RAM, using routinely collected surveillance data from three national networks in Switzerland, France, and England [17]. They trained a logistic regression model using 46,320 colorectal surgery records from 2007 to 2017 and compared it with the previous model developed by Mu et al. In their dataset, the new model, with an AUROC of 0.65, outperformed the model developed by Mu et al. Their model was easy to use but was limited to colorectal surgery only. Meanwhile, in the absence of a high-accuracy RAM, van Walraven and Musselman developed a logistic regression model based on 362,431 clinical data points from the National Surgical Quality Improvement Program [18]. The AUROC of this model reached 0.80. However, it required the users to provide large amounts of medical history information, such as ASA score, NNIS risk index, tumor history, medication history, and operation history. These variables are not always well structured in many electronic medical record (EMR) systems, and without the support of automatic extraction, completing evaluations based on this model undoubtedly consumed large amounts of time. Therefore, a gap still exists between current preoperative RAMs and the ideal RAM, which is generalized, accurate, and easy to use or deploy.

With the widespread use of hospital information systems and EMR systems in medical institutions, we can now use massive clinical data to build RAMs. In addition to structured data, we can also use natural language processing and deep learning technology to parse semantics from unstructured clinical text data and save time for manual extraction of text information. Many researchers have developed surveillance models using data from EMRs to automatically help infection control staff efficiently identify SSIs among massive medical records and have achieved high accuracy [19-22]. However, these models used not only preoperative information but also surgical, postoperative, and antibiotic information. Thus, they cannot be used to guide preoperative intervention.

To fill in the gap, we aimed to provide a solution in the form of the Artificial intelligence–based Multimodal Risk Assessment Model for Surgical site infection (AMRAMS) for inpatients undergoing operations using routinely collected data from the EMR system of a general hospital in China. We believed that structured data, such as patient demographics and preoperative lab results, and free-text data, such as preoperative notes that record diagnoses and scheduled surgical information, would both help to identify high-risk patients. Thus, we planned to combine various machine learning, deep learning, and semantic representation technologies; validate the discriminations of multimodal implementations internally and externally; and compare them with the NNIS risk index score. ﻿We tested the following hypotheses: (1) AMRAMS, with various implementations, would more accurately identify high-risk patients than the old-fashioned NNIS risk index is capable of doing, (2) semantic information from preoperative notes would improve the model performance, and (3) deep learning implementations would outperform conventional machine learning implementations.

## Methods

### Source of Data

The Rui Jin Hospital, Luwan Branch, affiliated with the Shanghai Jiao Tong University School of Medicine, is a nonprofit academic medical center based in Huangpu District, Shanghai, China. The hospital has a total of 526 beds, of which 89 are in general surgery, 33 are in gynecology, 27 are in orthopedics, and 38 are in urology. The surgical staff performs more than 4000 operations annually. About 300 of these cases are emergency patients. We retrieved inpatient records that each had only one operation record during the hospital stay and a discharge record between January 1, 2014, and June 30, 2019, from the EMR system of Rui Jin Hospital, Luwan Branch. We used data from before July 1, 2018, as the development set for model training, hyperparameter tuning, and internal validation; we used the remaining data as the test set for external validation. The data usage of patient records for this study had been reviewed and approved by the ethics committee of the Rui Jin Hospital, Luwan Branch.

### Participants and Features

We included adult patients only and excluded patients under the age of 18 years, patients with missing operation information (ie, timestamp of operation, whether or not theirs was an emergency operation, and type of anesthesia), and patients with missing demographic information (ie, gender and age).

We used both structured and unstructured preoperative clinical data from the EMR as our modeling features for this study. *Preoperative* was defined as the last record before the timestamp of the operation start time. Structured data included the following:

1. Patient demographics: age (years), gender (male or female), body height (cm), body weight (kg), and type of insurance (insured or noninsured).
2. Routine blood examination: white blood cell count (number × 10^9^/L), proportion of neutrophils (%), proportion of lymphocytes (%), proportion of monocytes (%), proportion of eosinophils (%), proportion of basophils (%), lymphocyte count (number × 10^9^/L), monocyte count (number × 10^9^/L), eosinophil count (number × 10^9^/L), red blood cell count (number × 10^12^/L), hemoglobin concentration (g/L), mean corpuscular volume (fL), mean corpuscular hemoglobin (g/L), mean corpuscular hemoglobin concentration (g/L), and platelet count (number × 10^9^/L).
3. Coagulation function examination: prothrombin time (sec), international normalized ratio, fibrinogen concentration (g/L), activated partial thromboplastin time (sec), thrombin time (sec), and d-dimer concentration (mg/L).
4. Liver and kidney function examination: total bilirubin concentration (μmol/L), direct bilirubin concentration (μmol/L), indirect bilirubin concentration (μmol/L), total bile acid concentration (μmol/L), alanine transaminase concentration (IU/L), aspartate aminotransferase concentration (IU/L), total protein concentration (g/L), albumin concentration (g/L), urea nitrogen concentration (mmol/L), creatinine concentration (μmol/L), uric acid concentration (μmol/L), and blood glucose concentration (mmol/L).
5. Plasmic electrolyte examination: potassium concentration (mmol/L), sodium concentration (mmol/L), calcium concentration (mmol/L), phosphorus concentration (mmol/L), and magnesium concentration (mmol/L).
6. Structured data elements from admission notes: current smoking status (true or false) and marital history (married, unmarried, or divorced).
7. Structured data elements from preoperative notes: emergency operation (true or false) and type of anesthesia (general anesthesia, total intravenous anesthesia, spinal anesthesia, epidural anesthesia, nerve block, or local anesthesia).
8. Preoperative LOS: the number of inpatient days between admission and operation.

Unstructured data included the free-text portion of the preoperative notes. Preoperative notes usually contain descriptions about preoperative diagnosis, operation name, indication, complications, and preventive measures. Table MA1-1 in Multimedia Appendix 1 shows two examples of preoperative notes from the development set.

### Outcome

According to the No. 48 Decree issued by the Ministry of Health of the People’s Republic of China in 2006 [23], the infection prevention and control department of the hospital should be responsible for the regular surveillance, analysis, and feedback on the epidemic situation of SSIs and their related risk factors. SSIs includes superficial incisional infection, deep incisional infection, and organ-space infection. In the Rui Jin Hospital, Luwan Branch, the staff of the infection prevention and control department manually identify SSIs via patient chart reviews and collect mandatory data for hospital administrators and government reporting after patient discharge. In this study, all the included patient records were reviewed by the infection prevention and control department. We categorized patient records with SSI identifications reported by the infection prevention and control department as positive samples. Likewise, we categorized patient records without SSI identifications as negative samples.

### Data Preprocessing

In this study, we used routinely collected clinical data from the EMR system. Thus, outliers and missing data were common and inevitable. For outlier adjustment, we first discarded patient records with invalid age values (eg, age >120 years old). To detect the outliers, we implemented a two-stage algorithm based on the random-effects model adjusted for age and sex proposed by Welch et al [24] for all continuous features. We used an absolute standardized residual of more than 5 as a cutoff for outlier detection and manually reviewed all the outliers suggested by the model. If the outliers violated medical knowledge, we tried to correct the values via a chart review. If no information could be gained from the chart review, we considered the outliers as missing values. For missing data, we generated missing-value indicators [25] (ie, binary dummy features indicating whether the values of the original features were missing) and conducted mean imputation for continuous features and mode imputation for binary or categorical features. To make the results of model validation truly reflect real-world performance, we performed outlier detection and adjustment only on the development set. For the convenience of model training and optimization, we performed one-hot encoding for all the binary or categorical features and feature normalization for all features.

### Model

#### Overview

Using the fastText algorithm, we first generated word embeddings based on a large Chinese corpus for further text-information encoding [26]. The fastText algorithm is an unsupervised neural network algorithm that learns distributional embeddings of semantic representation based on subword information for each word from the corpus. We then proposed both conventional machine learning methods and deep learning methods to predict the risk of SSI based on preoperative EMR data. Because we expected the distribution of the labels to be extremely imbalanced, we passed a positive sample weight (ie, the ratio of the number of non-SSIs to the number of SSIs) to the loss function (ie, cross-entropy) during the model training. We used the mini-batch gradient descent and backpropagation technique to update the parameters of the networks and set the AdamW algorithm as optimizer [27]. Furthermore, we used a random search method based on five-fold cross-validation and early stopping, if necessary, to find the optimized hyperparameters for each model.

#### Word Embedding

Our Chinese corpus contained approximately 4.1 GB of data from the Chinese Wikipedia, downloaded from the linguatools website [28], and approximately 96.9 MB of data from A-hospital, a Chinese medical Wikipedia website [29]. After we removed punctuations and numbers from the corpus, we used Jieba, version 0.41 [30], a Chinese text-segmentation tool, with a medical dictionary to segment the corpus into word sequences. Using the skipgram model of the fastText algorithm [31], we then trained 128-dimensional word embeddings using the preprocessed word sequences. We set the minimum size of the subwords as two and the maximum size as five. We left the other parameters at their default values.

#### Conventional Machine Learning Method

The conventional machine learning methods analyzed in this study included LASSO (least absolute shrinkage and selection operator) logistic regression with L1 penalty, random forest, and gradient boosting decision tree (GBDT), implemented by the XGBoost framework [32]. Because these models can be trained only by using tabular data, we first encoded the texts of the preoperative notes into the text embeddings. We segmented each text into a word sequence using Jieba and transformed it into a sequence of word embeddings, which is represented as follows:

*T* = [*t*_1_,*t*_2_,*t*_3_,...,*t_n_*] (**1**)

Here, *t_i_* is a 128-dimensional word-embedding vector of the *i*-th word in a sequence of length *n*, and *T* is an *n*-by-128 embedding matrix. We then pooled the embedding matrix into a 128-dimensional vector using the max-pooling method, which took the maximum value among the *n* words for each feature of the word embeddings. We concatenated the pooled vectors with the structured feature vectors and fed them into the four models for training.

#### Deep Learning Methods

**Figure 1:**
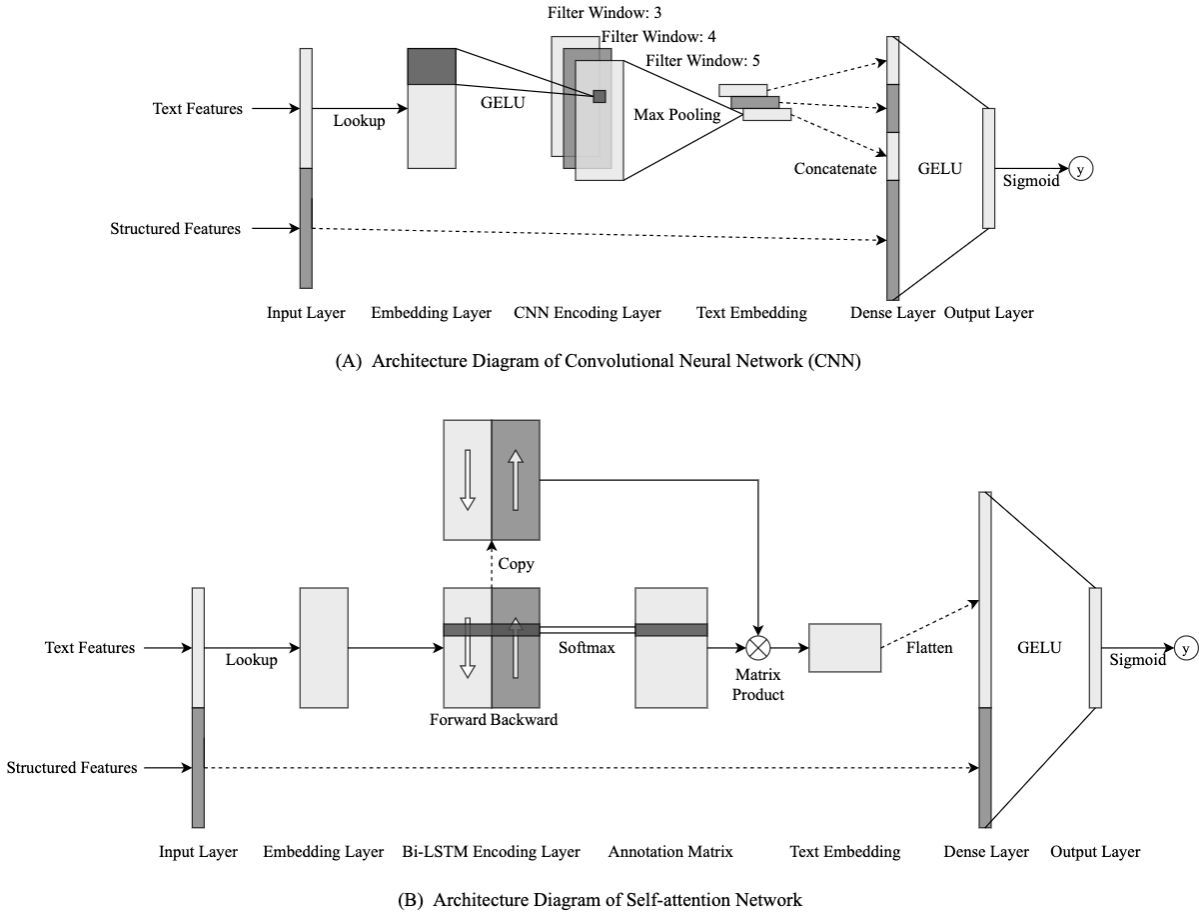
Deep learning network architecture diagrams. Bi-LSTM: bidirectional long short-term memory; GELU: Gaussian Error Linear Unit.

The deep learning methods analyzed in this study included a convolutional neural network (CNN) and a self-attention network. In this study, we used CNN and self-attention structures to encode text information on an end-to-end basis. Figure 1 shows the architecture of both models. Before being fed into the models, text data were transformed into *n*-by-128 embedding matrix *T*. Here, *n* was the padding length decided by the upper boundary of the 1.5-IQR rule based on the distribution of word sequence lengths in the development set. If the actual length of the sequence was less than *n*, we added zero-padding to the left side of the sequence. If the actual length was more than *n*, we tailored the left side of the sequence to suit the padding length.

For the CNN, we referred to the structure proposed by Kim [33] and applied convolutional kernel operation on the embedding matrix *T*, which is represented as follows:

*c^j^_i_* = *GELU*(*w^j^_c_* ∙ *t_i:I_*_+_*_h_*_–1_ + *b*) (**2**)

Here, *c^j^_i_* is the output value of the *j*-th convolutional channel of filter window *i*, *t_i:i_*_+_*_h_*_–1_ is the word-embedding sequence from the *i*-th word to the (*i* + *h* – 1)-th word, *h* is the size of the filter window, *w^j^_c_* is the weight vector for each word embedding in the filter window of the *j*-th channel, *b* is the bias item, and *GELU* (Gaussian Error Linear Unit) [34] is the active function; the original paper used *ReLU* (Rectified Linear Unit). The filter window slides from the first word to the last one. Let *m* represent the number of channels for the convolutional kernel, and let *n* represent the length of the text; then we have an (*n* – *h* + 1)-by-*m*

convolutional feature matrix:

*C* = [*c*_1_,*c_2_*,*c*_3_,...,*c_n_*_–_*_h_*_+1_] (**3**)

We used filter windows of three, four, and five; generated three convolutional feature matrices; applied max-pooling operation on these matrices; and concatenated the three pooled vectors and the structured feature vector together. The entire vector was then passed into a fully connected dense layer using *GELU* as an active function and, finally, a *sigmoid* output layer.

For the self-attention network, we referred to the structure proposed by Lin et al [35]. The embedding matrix *T* was first passed into a bidirectional long short-term memory (Bi-LSTM) layer. Let *m* represent the number of the hidden units for both forward and backward long short-term memory (LSTM), and let *n* represent the length of the word sequence. We obtained an *n*-by-2*m* hidden state feature matrix:

*H* = [*h*_1_,*h*_2_,*h*_3_,...,*h_n_*] (**4**)

We then generated an attention matrix based on the hidden state feature matrix. According to the original paper, this process was similar to passing the hidden state feature matrix into two bias-free, fully connected layers using *tanh* as the first active function and *softmax* as the second function:

*A* = *softmax*(*W*_1_ ∙ *tanh*(*W*_2_ ∙ *H^T^*)) (**5**)

Here, *W*_2_ is a *d*_1_-by-2*m* weight matrix, where *d*_1_ is the hidden unit number of the first layer and *W*_2_ is a *d*_2_-by-*d*_1_ weight matrix, where *d*_2_ is the hidden unit number of the second layer. We obtained a *d*_1_-by-2*m* text-embedding matrix by using the following:

*M* = *A* ∙ *H* (**6**)

We flattened the embedding matrix into a *d*_1_ × 2*m*-dimensional vector, concatenated it with the structured feature vector, and passed the entire vector into the dense layer.

### Evaluation

We evaluated the discrimination capacities of the conventional models, with or without text embeddings, and of the deep learning models. We assessed internal validity using a bootstrapping procedure of 100 iterations based on the development set. In each iteration, we trained the model using the sample data (ie, sampling with replacement) and tested the AUROC on the out-of-bag data; the data were not sampled in the iteration. We calculated the average AUROC and 95% CI for each model and performed paired-sample *t* tests to compare the performance among the models.

To obtain a realistic estimation of the model performance, we assessed external validity on the holdout test set. We trained the models using the entire development set, tested the full training performances on the test set, and compared them with the performance of the NNIS risk index scored by surgeons before an operation. Furthermore, we calculated the sensitivity and specificity based on the test result and decided on the cutoff point using Youden’s index method [36].

We used scikit-learn, version 0.22.1, via Python, version 3.7.4 (Python Software Foundation), to build the LASSO model and random forest model; we used XGBoost, version 0.9, via Python to build the GBDT model; and we used PyTorch, version 1.4.0, via Python to build the CNN and self-attention network. We performed all the statistical analyses using R, version 3.6.1 (The R Foundation), and considered a two-sided *P* value of <.05 as statistically significant.

## Results

### Patient Characteristics

**Figure 2:**
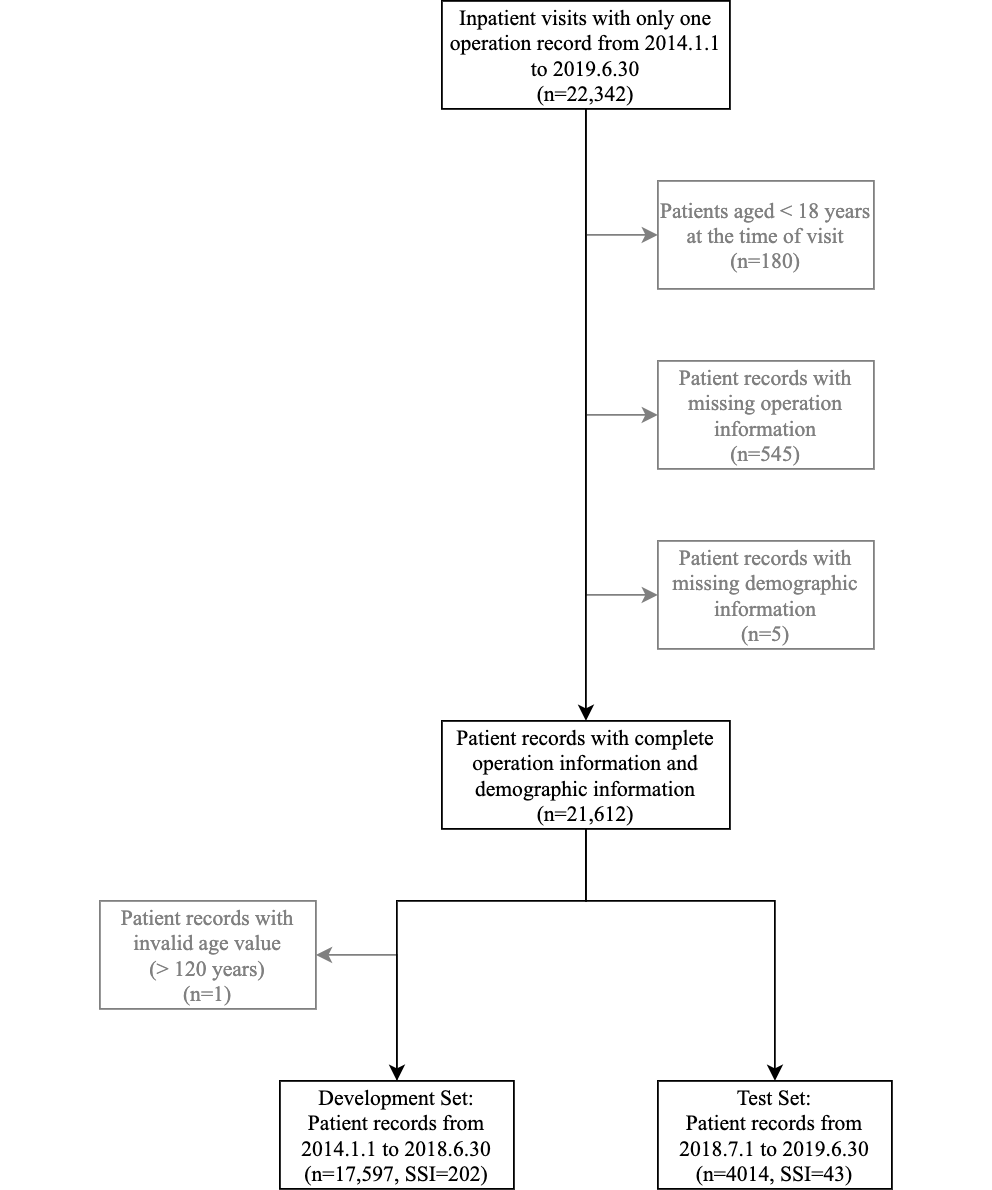
Flowchart of the selection process for patient records. Gray boxes show records that were excluded due to patients not meeting inclusion criteria and records containing outliers or missing data. SSI: surgical site infection.

We included a total of 21,611 inpatient records from January 1, 2014, to June 30, 2019. Of these records, 13,293 (61.51%) were from female patients and 8318 (38.49%) were from male patients with a median age of 54.3 years (IQR 44-65); 8375 (38.75%) were from the department of general surgery; 5903 (27.31%) were from the department of urology; 4649 (21.51%) were from the department of gynecology; and 2684 (12.42%) were from the department of orthopedics. According to the distributions of the International Classification of Diseases, Tenth Revision (ICD-10) code of operation and diagnosis that were retrieved after patient discharge, the patients received surgical treatment mainly for genitourinary system diseases, neoplasms, and digestive system diseases; the main types of operations were urinary system surgery, digestive system surgery, female reproductive system surgery, and endocrine system surgery. Overall, the incidence of SSIs in our dataset was 1.13% (244/21,611). The assigned sample size of the development set was 17,597 and that of the test set was 4014. The missing-data rates of the included variables ranged from 0% to 70.9% in the development set and from 0% to 72.8% in the test set. The variables with missing-data rates of more than 20% came from liver and kidney function examination, plasmic electrolyte examination, and d-dimer measurement. A slight difference was observed in the missing-data rate of each variable between the development set and the test set. Among them, the variables with the largest differences in the missing-data rates came from the electrolyte examinations (ie, calcium, phosphorous, and magnesium), with the rate differences reaching 8.0%. Figure 2 shows the selection process for the patient records and Table MA1-2 in Multimedia Appendix 1 shows the patient characteristics. We released a portion of the raw data in Multimedia Appendix 2, and the data dictionary of the raw data is located in the Data Description section of Multimedia Appendix 1.

### Hyperparameters and Training

We selected the optimal hyperparameters for each model based on the results of five-fold cross-validation. For LASSO, we used an L1 penalty of 0.01 when using text embeddings and 0.003 when not using text embeddings. For random forest with text embeddings, we used 300 trees, a maximum depth of 18, and maximum features of 0.6. For random forest without text embeddings, we used 1000 trees, a maximum tree depth of 4, and maximum features of 0.6. For GBDT with text embeddings, we used a learning rate (η) of 0.01, a maximum tree depth of 24, a subsample of 0.6, a column sample of 0.65, a gamma of 0.3, and 61 iterations. For GBDT without text embeddings, we used a learning rate (η) of 0.003, a maximum tree depth of 4, a subsample of 0.65, a column sample of 0.8, a gamma of 0, and 132 iterations. For the CNN, we used a learning rate (η) of 0.0001; an L2 penalty of 3; a word-embedding layer dropout rate of 0; CNN filter windows of three, four, and five with 256 feature maps (ie, channels) each; a dropout rate of 0.35; a fully connected layer with 128 feature maps; a dropout rate of 0.5; and 18 epochs. For the self-attention network, we used a learning rate (η) of 0.0001, an L2 penalty of 0.03, a word-embedding layer dropout rate of 0.5, a Bi-LSTM with 256 feature maps (ie, hidden nodes) each, a dropout rate of 0.45, an attention network with 256 feature maps (ie, hidden nodes) on the first layer and 64 for the second layer, a fully connected layer with 128 feature maps, a dropout rate of 0, and 19 epochs. We set the padding length for deep learning to 244. Hyperparameters not mentioned in this section were left at their default values.

### Model Performances

**Figure 3:**
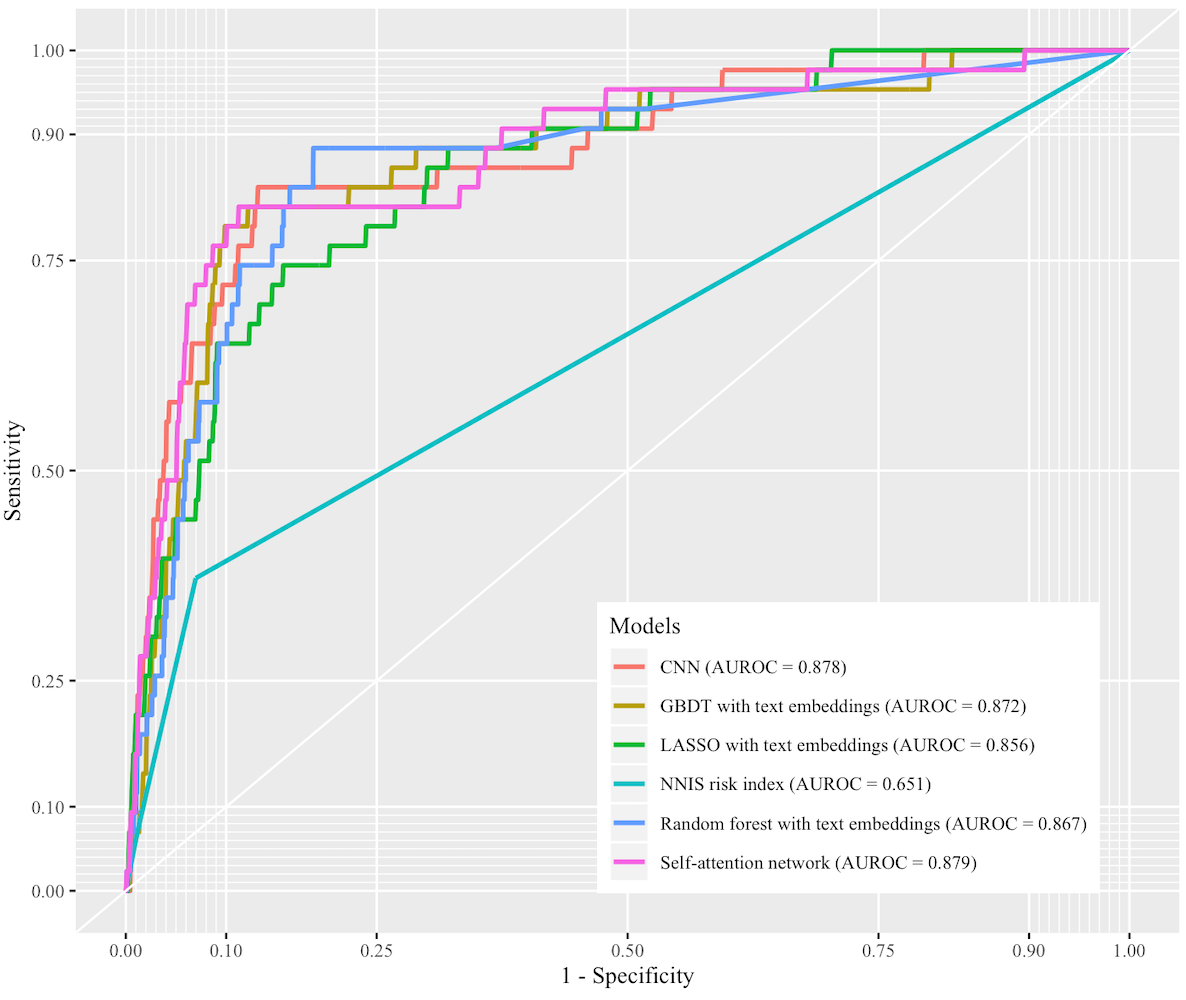
The receiver operating characteristic (ROC) curves of the top five models based on full training and National Nosocomial Infections Surveillance (NNIS) risk index. AUROC: area under the receiver operating characteristic curve; CNN: convolutional neural network; GBDT: gradient boosting decision tree; LASSO: least absolute shrinkage and selection operator.

Table 1 lists the performances of the models in terms of both internal and external validation, and Figure 3 shows the receiver operating characteristic (ROC) curves of the top five models based on full training and NNIS risk index. For internal validation, CNN yielded the highest mean AUROC of 0.889 (95% CI 0.886-0.892), and the paired-sample *t* test (see Multimedia Appendix 1, Table MA1-3) revealed statistically significant advantages (*P*<.001) compared with the other models. The self-attention network yielded the second-highest mean AUROC of 0.882 (95% CI 0.878-0.886). However, the AUROC of the self-attention network was only numerically higher than the AUROC of the third-best model—GBDT with text embeddings (mean AUROC 0.881, 95% CI 0.878-0.884)—and did not exhibit statistical significance (*P*=.47). The AUROCs of the machine learning models using text embeddings were statistically higher than the AUROCs of the models not using text embeddings (*P*<.001). For external validation, the self-attention network yielded the highest AUROC of 0.879. CNN was the second-best model (AUROC 0.878), and GBDT with text embeddings was the third-best model (AUROC 0.872). The NNIS risk index scored by surgeons had an AUROC of 0.651, which was remarkably lower than that of any other model in our study. Based on the external validation, we could still observe a trend with the text embeddings improving the model performances in the external validation. All the models had lower AUROC scores in internal validation than in external validation (ie, mean AUROC).

**Table 1:** Model performances.

Model and text embedding		Area under the receiver operating characteristic curve (AUROC)		Sensitivity^a^ (full training)	Specificity^a^ (full training)
		Bootstrapping, mean (95% CI)	Full training		
**Least absolute shrinkage and selection operator (LASSO)**					
	With text embedding	0.870 (0.867-0.874)	0.856	0.744	0.844
	Without text embedding	0.856 (0.852-0.860)	0.816	0.674	0.842
**Random forest**					
	With text embedding	0.877 (0.873-0.880)	0.867	0.884	0.813
	Without text embedding	0.846 (0.842-0.850)	0.772	0.558	0.871
**Gradient boosting decision tree (GBDT)**					
	With text embedding	0.881 (0.878-0.884)	0.872	0.791	0.902
	Without text embedding	0.838 (0.834-0.843)	0.782	0.605	0.858
Convolutional neural network (CNN)		0.889 (0.886-0.892)	0.878	0.837	0.869
Self-attention		0.882 (0.878-0.886)	0.879	0.814	0.888
National Nosocomial Infections Surveillance (NNIS) risk index		N/A^b^	0.651	0.372	0.930

^a^The optimal cutoff point was identified using Youden’s index method.

^b^N/A: not applicable.

### Feature Analysis

Both deep learning models—CNN and self-attention network—performed better than other models in our validations. However, the deep learning models were black boxes and hard to explain. To further explore the correlations between the selected features and the occurrence of SSIs, we conducted a population-level feature analysis for the structured features and a case-level analysis for the text embeddings. The population-level analysis explored the correlations by comparing the normalized coefficient for each feature from LASSO without text embeddings; the coefficients were based on the data after normalization. For case-level analysis, we referenced the idea from local interpretable model-agnostic explanations [37]. For each case, we fixed the structured features and generated new word sequences by randomly removing words from the raw sequence and dummy binary vectors that indicated whether the word in a certain position was removed or not. We generated 10,000 new sequences for each case, combined them with the structured features, passed them to the deep learning models, and obtained prediction scores. We then fitted a LASSO regression model—with an L1 penalty of 0.01—that uses dummy binary vectors as features and prediction scores as targets. The coefficients of the LASSO regression model indicated the relative contributions of the words to the prediction scores in a case.

**Figure 4:**
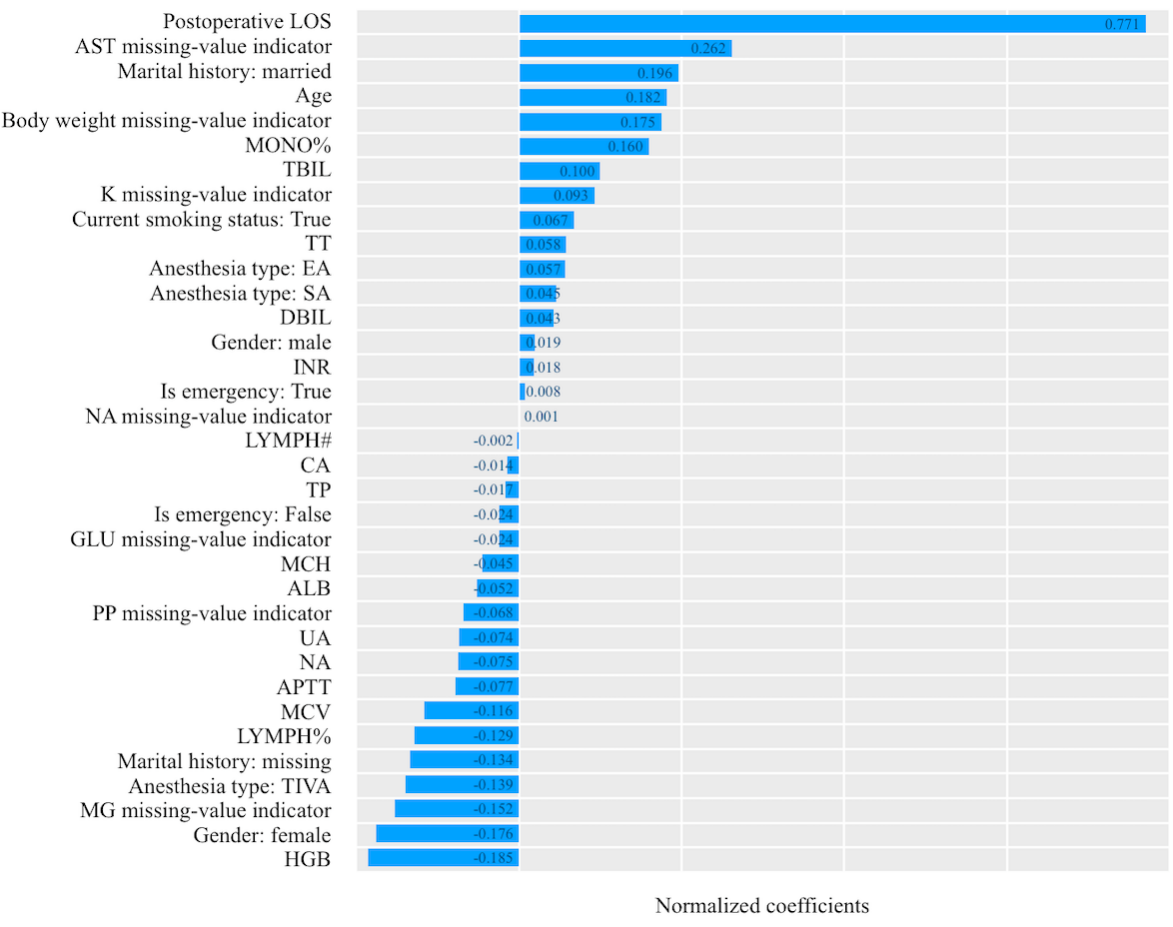
The normalized coefficients of the features in the LASSO (least absolute shrinkage and selection operator) model without text embeddings. ALB: albumin; APTT: activated partial thromboplastin time; AST: aspartate aminotransferase; CA: calcium; DBIL: direct bilirubin; EA: epidural anesthesia; GLU: blood glucose; HGB: hemoglobin; INR: international normalized ratio; K: potassium; LOS: length of stay; LYMPH: lymphocyte; MCH: mean corpuscular hemoglobin; MCV: mean corpuscular volume; MG: magnesium; MONO: monocyte; NA: sodium; PP: phosphorus; SA: spinal anesthesia; TBIL: total bilirubin; TIVA: total intravenous anesthesia; TP: total protein; TT: thrombin time; UA: uric acid.

Figure 4 shows the features with nonzero coefficients and their coefficients for the population-level analysis. We could observe that preoperative LOS, marital history, anesthesia type, gender, age, results of routine blood examination, coagulation function examination, and many missing-value indicators had remarkable impacts on the model. Among them, patients with prolonged preoperative LOS, patients with missing AST results, married patients, older patients, and patients with missing body weight information had higher risks of SSI. Patients with higher hemoglobin, female patients, patients with missing magnesium results, patients that had received total intravenous anesthesia, and patients with missing marital histories had lower risks of SSI.

**Figure 5:**
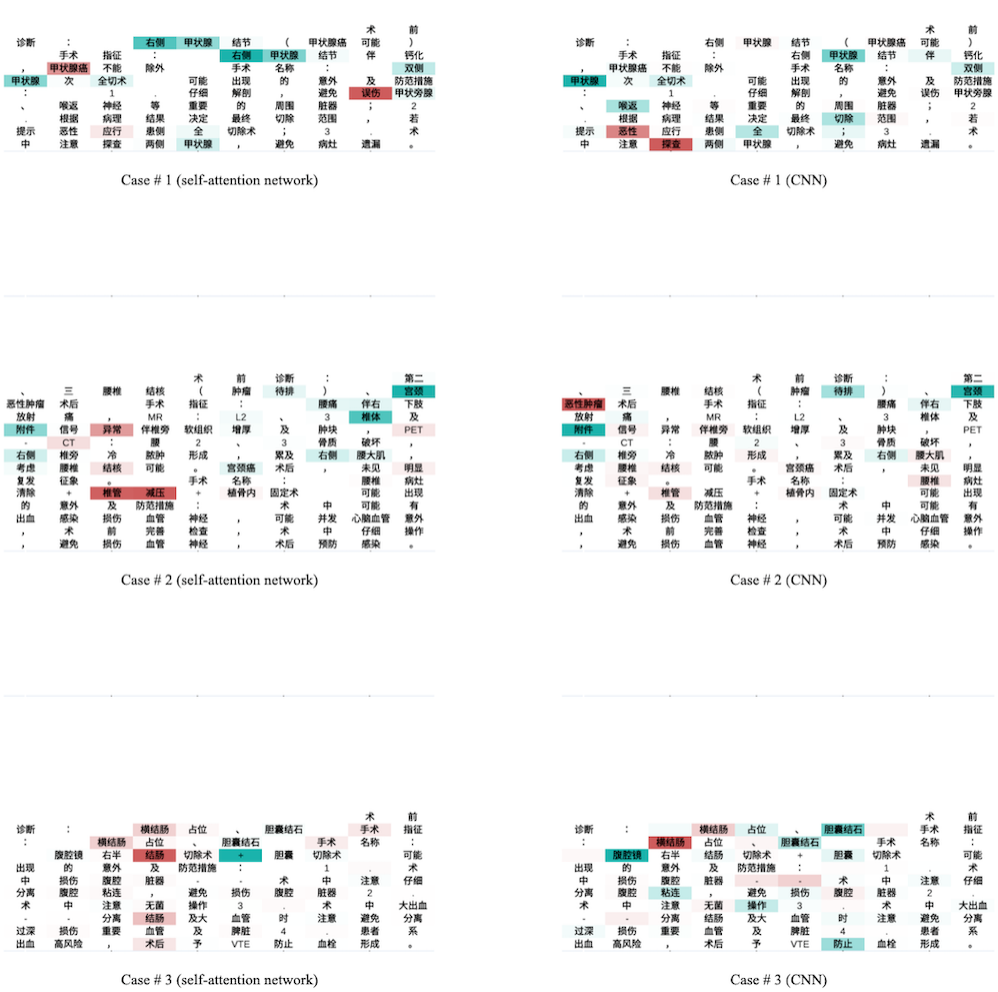
The heatmaps of the word contributions on three preoperative note cases. CNN: convolutional neural network.

Figure 5 shows the heatmaps of the word contributions to the CNN and attention prediction scores for three preoperative note cases (see Table MA1-1 in Multimedia Appendix 1 for the full-text translation), with green being a negative coefficient (ie, protective factor) and red being a positive coefficient (ie, risk factor). The deeper the color, the higher the absolute value. Among the three cases, we could observe that terms like “甲状腺 (thyroid),” “宫颈 (uterine neck),” “附件 (accessory),” “椎体 (centrum),” “腹腔镜 (laparoscope),” and “胆囊结石 (gallstone)” were associated with lower risk of SSI, and terms like “甲状腺癌 (thyroid cancer),” “恶性 (malignant),” “结核 (tuberculosis),” “恶性肿瘤 (malignant tumor),” “结肠 (colon),” and “横结肠 (transverse colon)” were associated with higher risk of SSI.

## Discussion

### Principal Findings

In this study, we found that SSI RAMs based on clinical data from an EMR system and modern machine learning techniques could identify high-risk patients more accurately than the old-fashioned NNIS risk index is capable of doing. Notably, the vectorial embedding of preoperative notes, whether generated using a simple max-pooling method or using a deep learning method, improved the performance of the model further without any handcrafted feature engineering. The multimodal deep learning models that produced end-to-end feature representations automatically through convolutional kernels, LSTM, or attention mechanisms outperformed the traditional machine learning models, such as LASSO, random forest, or GBDT. Thus, our AMRAMS using a CNN or a self-attention network could replace the NNIS risk index in providing personalized guidance for the preoperative intervention of SSI. At the same time, our study provided an easy-to-implement solution to building a multimodal RAM for similar scenarios based on both structured and Chinese text data. Because we used routinely collected preoperative data only, such as the results of routine blood tests and clinical notes, additional manual data collection and clinician evaluation was no longer necessary for achieving high accuracy.

Many factors could explain the advantages of the deep learning AMRAMS. First, we used more objective quantitative features of the patients. The NNIS risk index contained only three elements, of which the ASA score was a subjective feature provided by anesthesiologists. The model developed by Mu’s team, on the other hand, utilized 12-15 features and included the ASA score [16]. The model developed by Grant’s team utilized seven features and also included the ASA score [17]. The model developed by van Walraven and Musselman utilized 53 features and included not only subjective features, such as the ASA score, the NNIS risk index score, and dyspnea evaluation, but also 33 manually extracted variables from the medical history of the patient [18]. Meanwhile, our model included 47 objective features, among which age, gender, body weight, anesthesia type, emergency operation, preoperative hemoglobin, glucose, and LOS have proven to be related to the occurrences of SSI [12,38,39]. All these features were the results of routine preoperative blood tests and were automatically extracted from the EMR system using SQL (Structured Query Language) query script.

Second, we used sufficient information about the operative procedures and risk prevention from the preoperative notes via the fastText embeddings and the network structures. Many studies on English text classification have demonstrated that machine learning using fastText embeddings had better performance than those of the algorithms using bag-of-words, n-grams, TF-IDF (term frequency–inverse document frequency), or word2vec embeddings [26,40]. The semantics of many words, especially in the Chinese language, depend on the subwords or characters they contain. The fastText algorithm generated the semantic embeddings based on the internal structures of words, which best suit the characteristics of natural language. Moreover, our deep learning models enabled end-to-end learning: both fastText embeddings and hidden nodes of the network could be further fine-tuned simultaneously according to the specified targets during the learning process. To encode text-level semantics, we tried both convolutional kernel and attention mechanisms. These network structures could automatically represent n-grams and long-term dependency information, which helped the deep learning models gain better performance than those of conventional machine learning models (ie, logistic regression, naïve-Bayes, and support-vector machine) trained using the top of max-pooling embeddings [33,35]. Because of the complexity of the deep learning models, we were not able to precisely identify the decision mechanisms of the text semantics. However, according to the heatmaps of case-level word contribution, the potentially essential keywords helped identify SSIs in the form of distributed representation without any other handcrafted feature engineering or manual feature extraction. Potentially essential keywords included those suggesting endoscopic surgery, such as “laparoscope,” and “gallstone”; those suggesting clean surgery, such as “thyroid” and “centrum”; those suggesting colon surgery, such as “colon” and “transverse colon”; and those suggesting complex operation and prolonged operation time, such as “malignant,” “tuberculosis,” and “malignant tumor.”

Third, we tried many algorithmic techniques to avoid overfitting. For example, we used the L2 penalty, dropout, and early-stopping techniques. These techniques ensured the generalization ability of the model on different patient data to a certain extent.

Although we verified the effectiveness of the deep learning AMRAMS through both internal and external verification, many limitations still exist. The first limitation came from the training labels. The follow-up period of SSI by the infection prevention and control department was limited only to the hospitalization, which meant some of the SSIs that occurred after discharge might have been ignored. Although surgeons would conduct a careful examination of the incision before patient discharge, the occurrence of SSIs outside secondary care could not be completely eliminated. This bias would cause our model to underestimate the risk of patients developing SSIs.

The second limitation came from the patient population. Our dataset came from one medical site, and the time span of data collection was about 5 years, in which changes in patient population distribution, surgical procedures, and SSI prevention education and measures were inevitable. In our study, the internal validation results of the models were not completely consistent with the external verification results, which implied this point. If our model was to be applied to clinical practice, regular validation and update would be necessary.

The third limitation came from the missing values. We observed that many variables in our dataset had high missing rates, and many missing-value indicators contributed greatly to the model. In general, the missing data in the EMR system were not missing at random and were caused mainly by two reasons: inability to perform the measurement or a lack of indication to perform the measurement. For example, missing body weight information might indicate that the patient was unable to stand upright (eg, paralyzed) in order to measure the weight, whereas missing blood tests and liver function tests might suggest that the patient was healthy and young. In our study, we were not able to evaluate the potential influence of the missing data, because speculating the reason behind each missing value was complex and trivial. From a perspective of research, we could try to model the probability of missingness using other observed variables and conduct sensitivity analyses, which stimulate various missing patterns based on the predicted probability distributions, to evaluate the influences of missing data in our future studies. However, the ideal solutions to missing-data problems are still improving data quality and integrity in EMRs or developing less-biased imputation methods based on the patterns of the missing values in the patient records, the conditions of the patients, and the behaviors of the physicians.

The fourth limitation came from the models. We did not observe the attention mechanisms on the top of Bi-LSTM providing a great benefit over the convolutional kernels as claimed by Lin et al in their paper [35], probably because of the limited sizes of the training samples relative to the model parameters. Thus, we considered both self-attention and CNN as the best solutions in our current task. Because of the limitations of computing resources (ie, GPU [graphics processing unit] instances), we did not apply other state-of-the-art language models, such as BERT (bidirectional encoder representations from transformers) and its derivatives [41,42], to encode text information.

The fifth limitation came from the feature analysis. In this study, we only explored the correlations between the selected features and the occurrences of SSIs via the LASSO models, with detailed epidemic investigations and causal inferences remaining beyond the scope of this study. Moreover, because the LASSO models were trained using incomplete data and were not adjusted for potential confounders, the statistical inferences would be biased and the results would be hard to explain.

Future studies will focus on four points. First, we will try various new language models with deep transformers; encode various text information types, such as admission records, progress notes, and surgical records; and evaluate the models’ performance. Second, we will confirm the effectiveness of our AMRAMS among multiple medical sites. Third, we will embed the AMRAMS into the EMR system and evaluate whether it can ultimately help reduce the occurrence of SSIs and optimize medical decision making. Fourth, our multimodal RAM solution could be validated for many other similar scenarios, such as syndromic or notifiable disease surveillance, adverse event monitoring, or ICD-10 coding support, in which both structured and free-text features would contribute to the judgement of final outcomes.

### Conclusions

Our artificial intelligence–based multimodal risk assessment models for SSI based on EMR data and deep learning methods had significant advantages in terms of accuracy, compared with other conventional machine learning methods and the NNIS risk index. The semantic embeddings of clinical notes, whether generated using a simple max-pooling method or a deep learning method, improved the model performance further without any handcrafted feature engineering. Our models could replace the NNIS risk index to provide personalized guidance for the preoperative intervention of SSI. Through this case, we offered an easy-to-implement solution for building multimodal RAMs for similar scenarios, based on both structured and free-text data. Future studies should validate the generalization, reproducibility, and clinical impact in other medical settings.

## Appendix

Multimedia Appendix 1 The data description of the raw data and additional tables.

Multimedia Appendix 2 A portion of the patient records raw data.

## Abbreviations

AMRAMS: Artificial intelligence–based Multimodal Risk Assessment Model for Surgical site infection
ASA: American Society of Anesthesiologists
AUROC: area under the receiver operating characteristic curve
BERT: bidirectional encoder representations from transformers
Bi-LSTM: bidirectional long short-term memory
CNN: convolutional neural network
EMR: electronic medical record
GBDT: gradient boosting decision tree
GELU: Gaussian Error Linear Unit
GPU: graphics processing unit
HAI: health care–associated infection
ICD-10: International Classification of Diseases, Tenth Revision
LASSO: least absolute shrinkage and selection operator
LOS: length of stay
LSTM: long short-term memory
NHSN: National Healthcare Safety Network
NNIS: National Nosocomial Infections Surveillance
RAM: risk assessment model
ReLU: Rectified Linear Unit
ROC: receiver operating characteristic
SQL: Structured Query Language
SSI: surgical site infection
TF-IDF: term frequency–inverse document frequency
