# Supplementary material for: Artificial Intelligence–Based Multimodal Risk Assessment Model for Surgical Site Infection (AMRAMS): Development and Validation Study
Source: JMIR Med Inform. 2020 Jun 15;8(6):e18186. doi: 10.2196/18186 (PMC7325005; doi:10.2196/18186)
Supplement: Multimedia Appendix 1 [file medinform_v8i6e18186_app1.docx]

**Data description**

To make our study more transparent and reproducible to the interested researchers, we released a portion of the raw data (see Multimedia Appendix 2). However, due to the concern of patient privacy, we fixed the age of patients older than 89 to 300 and fixed the preoperative LOS more than 30 days to 300 days. Moreover, our hospital policy further discourages the sharing of the clinical notes across medical sites, and we moved the information of preoperative notes in the datasets. The details of the data elements are presented below.

| **Name** | **Description** | **Data type** | **Value Set** |
| --- | --- | --- | --- |
| age | Age (yr) | Numerical | / |
| body_height | Body height (cm) | Numerical | / |
| body_weight | Body weight (kg) | Numerical | / |
| WBC | White blood cell count (10^9/L) | Numerical | / |
| NEUT% | Proportion of neutrophil | Numerical | / |
| LYMPH% | Proportion of lymphocyte | Numerical | / |
| MONO% | Proportion of monocyte | Numerical | / |
| EOS% | Proportion of eosinophil | Numerical | / |
| BASO% | Proportion of basophil | Numerical | / |
| LYMPH# | Lymphocyte count (10^9/L) | Numerical | / |
| MONO# | Monocyte count (10^9/L) | Numerical | / |
| EOS# | Eosinophil count (10^9/L) | Numerical | / |
| RBC | Red blood cell count (10^12/L) | Numerical | / |
| HGB | Hemoglobin (g/L) | Numerical | / |
| MCV | Mean corpuscular volume (fl) | Numerical | / |
| MCH | Mean corpuscular hemoglobin | Numerical | / |
| MCHC | Mean corpuscular hemoglobin concentration (g/L) | Numerical | / |
| PLT | Platelet count (10^9/L) | Numerical | / |
| PT | Prothrombin time (sec) | Numerical | / |
| INR | International normalized ratio | Numerical | / |
| FIB | Fibrinogen (g/L) | Numerical | / |
| APTT | Activated partial thromboplastin time (sec) | Numerical | / |
| TT | Thrombin time (sec) | Numerical | / |
| DD | D-dimer (mg/L) | Numerical | / |
| TBIL | Total bilirubin (umol/L) | Numerical | / |
| DBIL | Direct bilirubin (umol/L) | Numerical | / |
| IBIL | Indirect bilirubin (umol/L) | Numerical | / |
| TBA | Total bile acid (umol/L) | Numerical | / |
| ALT | Alanine transaminase (IU/L) | Numerical | / |
| AST | Aspartate aminotransferase (IU/L) | Numerical | / |
| TP | Total protein (g/L) | Numerical | / |
| ALB | Albumin (g/L) | Numerical | / |
| GLU | Blood glucose (mmol/L) | Numerical | / |
| UREA | Urea nitrogen (mmol/L) | Numerical | / |
| CREA | Creatinine (umol/L) | Numerical | / |
| UA | Uric acid (umol/L) | Numerical | / |
| K | Potassium (mmol/L) | Numerical | / |
| NA | Sodium (mmol/L) | Numerical | / |
| CA | Calcium (mmol/L) | Numerical | / |
| PP | Phosphorus (mmol/L) | Numerical | / |
| MG | Magnesium (mmol/L) | Numerical | / |
| pre_opt_los | The number of inpatient days between admission and operation | Numerical | / |
| gender | Gender | Categorical | male; female |
| current_smoking_status | Current smoking status | Boolean | / |
| marital_history | Marital history | Categorical | divorced; married; unmarried; missing |
| insurance_type | Type of insurance | Categorical | insurance; non-insurance; missing |
| anesthesia_type | Type of anesthesia | Categorical | EA (epidural anesthesia); GA (general anesthesia); LA (local anesthesia); NB (nerve block); SA (spinal anesthesia); TIVA (total intravenous anesthesia) |
| is_emergency | Emergency operation | Boolean | / |
| label | SSI label | Categorical | 0 (non-SSI); 1 (SSI) |
| group | Dataset indicator | Categorical | dev (development set); te (test set) |

**Tables**

**Table MA1-1. Examples of the preoperative notes**

| **Case #** | **Chinese** | **English** |
| --- | --- | --- |
| 1 | 术前诊断：  右侧甲状腺结节（甲状腺癌可能）  手术指征：  右侧甲状腺结节伴钙化，甲状腺癌不能除外  手术名称：  双侧甲状腺次全切除术  可能出现的意外及防范措施：  1.仔细解剖，避免误伤甲状旁腺、喉返神经等重要的周围脏器；2.根据病理结果决定最终切除范围，若提示恶性应行患侧全切除术；3.术中注意探查两侧甲状腺，避免病灶遗漏。 | Preoperative diagnosis:  Right lobar thyroid nodule (probably thyroid carcinoma)  Operation indication:  Right lobar thyroid nodule with calcification, thyroid carcinoma cannot be ruled out  Operation name:  Bilateral subtotal thyroidectomy  Possible complications and preventive measures:  1. Carefully dissect the tissue and protect the important surrounding organs, such as parathyroid glands and recurrent laryngeal nerves. 2. Adjust the resection range according to (frozen) pathological diagnosis. If malignancy is indicated, total thyroidectomy should be carried out. 3. Carefully check both lobes of the thyroid gland to avoid misdiagnosis of lesions. |
| 2 | 术前诊断：  第二、三腰椎结核（肿瘤待排）、宫颈恶性肿瘤术后  手术指征：  腰痛伴右下肢放射痛，MR：L2、3椎体及附件信号异常伴椎旁软组织增厚及肿块，PET-CT：腰2、3骨质破坏，右侧椎旁冷脓肿形成，累及右侧腰大肌，考虑腰椎结核可能。宫颈癌术后，未见明显复发征象。  手术名称：  腰椎病灶清除+椎管减压+植骨内固定术  可能出现的意外及防范措施：  术中可能有出血感染损伤血管神经，可能并发心脑血管意外，术前完善检查，术中仔细操作，避免损伤血管神经，术后预防感染。 | Preoperative diagnosis:  L2 and L3 lumbar tuberculosis (tumor cannot be ruled out), operation history of cervical cancer  Operation indication:  Low back pain with radiation to the right leg. MR from the previously visited hospital: abnormal signal on L2 and L3 vertebral body and appendix; incrassated paravertebral soft tissue and mass were observed. PET-CT: bone destruction on L2 and L3; cold abscess formation was observed on the right paravertebral tissue with the right psoas major infiltration; lumbar tuberculosis was indicated. The patient received operation for cervical cancer and no signs of recurrence was observed.  Operation name:  Lumbar spine lesion debridement + spinal canal decompression + iliac bone graft fusion and internal fixation  Possible complications and preventive measures:  There may be bleeding, infection, injury of blood vessels and nerves during the operation, and maybe complicated with cardio-cerebrovascular accidents. Comprehensive examination before operation, careful operation to avoid injury of blood vessels and nerves, and prevention of infection after the operation are needed. |
| 3 | 术前诊断：  横结肠占位、胆囊结石  手术指征：  横结肠占位、胆囊结石  手术名称：  腹腔镜右半结肠切除术+胆囊切除术  可能出现的意外及防范措施：  1.术中损伤腹腔脏器--术中注意仔细分离腹腔粘连，避免损伤腹腔脏器。 2.术中注意无菌操作。 3.术中大出血--分离结肠及大血管时注意避免分离过深损伤重要血管及脾脏。 4.患者系出血高风险，术后予VTE防止血栓形成。 | Preoperative diagnosis:  Transverse colon mass, gallstones  Operation indication:  Transverse colon mass, gallstones  Operation name:  Laparoscopic right hemicolectomy + cholecystectomy  Possible complications and preventive measures:  1. Injury of abdominal organs during operation -- separate abdominal adhesions carefully and avoid injury of abdominal organs. 2. Conduct aseptic procedures. 3. Major bleeding during operation -- when separating the colon and large blood vessels, be careful not to separate too deep and avoid damaging the important blood vessels and the spleen. 4. The patient is at high risk of bleeding, prevent VTE after surgery. |

**Table MA1-2. The patient characteristics of the development data and test data**

| **Characteristic** | **Development set (n=17597)** | **Test set (n=4014)** |
| --- | --- | --- |
| SSI % (n) | 1.142% (201) | 1.071% (43) |
| Age (yr) |  |  |
| -Mean±SD (Median, IQR 25%-75%) | 54.346±15.060 (56.000, 44.000-65.000) | 53.937±14.783 (55.000, 43.000-64.000) |
| -Missing rate % (n) | 0.000% (0) | 0.000% (0) |
| Body height (cm) |  |  |
| -Mean±SD (Median, IQR 25%-75%) | 164.247±7.672 (163.000, 159.000-170.000) | 163.171±13.158 (163.000, 158.000-170.000) |
| -Missing rate % (n) | 0.148% (26) | 0.075% (3) |
| Body weight (kg) |  |  |
| -Mean±SD (Median, IQR 25%-75%) | 63.810±11.601 (62.500, 55.000-70.000) | 65.115±15.272 (63.000, 55.000-71.500) |
| -Missing rate % (n) | 1.460% (257) | 0.573% (23) |
| WBC (10^9/L) |  |  |
| -Mean±SD (Median, IQR 25%-75%) | 6.098±2.003 (5.770, 4.790-6.980) | 6.005±2.043 (5.680, 4.700-6.880) |
| -Missing rate % (n) | 12.127% (2134) | 12.905% (518) |
| NEUT% |  |  |
| -Mean±SD (Median, IQR 25%-75%) | 60.645±10.347 (60.400, 53.800-67.100) | 61.565±10.337 (61.250, 54.600-67.940) |
| -Missing rate % (n) | 12.121% (2133) | 12.905% (518) |
| LYMPH% |  |  |
| -Mean±SD (Median, IQR 25%-75%) | 30.620±9.395 (30.700, 24.500-36.900) | 29.919±9.439 (30.000, 23.800-36.400) |
| -Missing rate % (n) | 12.127% (2134) | 12.905% (518) |
| MONO% |  |  |
| -Mean±SD (Median, IQR 25%-75%) | 6.020±1.952 (5.800, 4.700-7.000) | 5.879±1.845 (5.700, 4.600-6.825) |
| -Missing rate % (n) | 12.121% (2133) | 12.905% (518) |
| EOS% |  |  |
| -Mean±SD (Median, IQR 25%-75%) | 2.331±2.024 (1.800, 1.040-3.000) | 2.282±2.153 (1.800, 1.000-2.900) |
| -Missing rate % (n) | 12.150% (2138) | 13.528% (543) |
| BASO% |  |  |
| -Mean±SD (Median, IQR 25%-75%) | 0.398±0.292 (0.300, 0.200-0.500) | 0.384±0.278 (0.300, 0.200-0.500) |
| -Missing rate % (n) | 12.150% (2138) | 13.528% (543) |
| LYMPH (10^9/L) |  |  |
| -Mean±SD (Median, IQR 25%-75%) | 1.790±0.702 (1.720, 1.370-2.130) | 1.719±0.685 (1.650, 1.300-2.070) |
| -Missing rate % (n) | 12.121% (2133) | 12.905% (518) |
| MONO (10^9/L) |  |  |
| -Mean±SD (Median, IQR 25%-75%) | 0.361±0.152 (0.330, 0.260-0.430) | 0.345±0.144 (0.320, 0.250-0.410) |
| -Missing rate % (n) | 12.121% (2133) | 12.905% (518) |
| EOS (10^9/L) |  |  |
| -Mean±SD (Median, IQR 25%-75%) | 0.138±0.130 (0.100, 0.060-0.174) | 0.132±0.138 (0.100, 0.057-0.170) |
| -Missing rate % (n) | 12.150% (2138) | 13.528% (543) |
| RBC (10^12/L) |  |  |
| -Mean±SD (Median, IQR 25%-75%) | 4.433±0.508 (4.440, 4.130-4.750) | 4.418±0.499 (4.420, 4.120-4.730) |
| -Missing rate % (n) | 12.121% (2133) | 12.905% (518) |
| HGB (g/L) |  |  |
| -Mean±SD (Median, IQR 25%-75%) | 130.737±16.712 (132.000, 122.000-141.000) | 131.292±16.708 (132.000, 122.000-142.000) |
| -Missing rate % (n) | 12.121% (2133) | 12.905% (518) |
| MCV (fl) |  |  |
| -Mean±SD (Median, IQR 25%-75%) | 87.426±6.468 (88.300, 84.500-91.400) | 89.849±5.582 (90.300, 87.500-93.100) |
| -Missing rate % (n) | 12.121% (2133) | 12.905% (518) |
| MCH |  |  |
| -Mean±SD (Median, IQR 25%-75%) | 29.547±2.517 (29.900, 28.800-30.800) | 29.759±2.328 (30.100, 29.000-31.100) |
| -Missing rate % (n) | 12.121% (2133) | 12.905% (518) |
| MCHC (g/L) |  |  |
| -Mean±SD (Median, IQR 25%-75%) | 338.112±18.197 (337.000, 328.000-347.000) | 331.005±12.677 (331.000, 324.000-338.000) |
| -Missing rate % (n) | 12.121% (2133) | 12.905% (518) |
| PLT (10^9/L) |  |  |
| -Mean±SD (Median, IQR 25%-75%) | 218.694±64.434 (213.000, 176.000-253.000) | 220.324±66.130 (215.500, 178.000-255.000) |
| -Missing rate % (n) | 12.121% (2133) | 12.905% (518) |
| PT (sec) |  |  |
| -Mean±SD (Median, IQR 25%-75%) | 11.565±1.244 (11.300, 10.800-12.000) | 10.639±1.251 (10.500, 10.100-11.000) |
| -Missing rate % (n) | 12.451% (2191) | 14.449% (580) |
| INR |  |  |
| -Mean±SD (Median, IQR 25%-75%) | 1.001±0.085 (0.990, 0.950-1.040) | 0.938±0.111 (0.930, 0.890-0.970) |
| -Missing rate % (n) | 12.434% (2188) | 14.449% (580) |
| FIB (g/L) |  |  |
| -Mean±SD (Median, IQR 25%-75%) | 2.840±0.851 (2.690, 2.270-3.200) | 2.768±0.810 (2.620, 2.220-3.140) |
| -Missing rate % (n) | 12.445% (2190) | 14.449% (580) |
| APTT (sec) |  |  |
| -Mean±SD (Median, IQR 25%-75%) | 26.634±4.136 (26.200, 23.800-29.100) | 25.542±3.664 (25.100, 23.100-27.400) |
| -Missing rate % (n) | 12.440% (2189) | 14.449% (580) |
| TT (sec) |  |  |
| -Mean±SD (Median, IQR 25%-75%) | 18.052±2.085 (18.300, 17.200-19.200) | 19.719±1.897 (19.600, 18.800-20.400) |
| -Missing rate % (n) | 12.457% (2192) | 14.524% (583) |
| DD (mg/L) |  |  |
| -Mean±SD (Median, IQR 25%-75%) | 0.162±0.247 (0.100, 0.100-0.100) | 0.168±0.170 (0.100, 0.100-0.200) |
| -Missing rate % (n) | 70.864% (12470) | 72.795% (2922) |
| TBIL (umol/L) |  |  |
| -Mean±SD (Median, IQR 25%-75%) | 13.132±7.601 (11.800, 9.000-15.300) | 12.446±6.547 (11.400, 8.700-14.700) |
| -Missing rate % (n) | 43.201% (7602) | 42.626% (1711) |
| DBIL (umol/L) |  |  |
| -Mean±SD (Median, IQR 25%-75%) | 2.667±3.210 (2.200, 1.700-3.000) | 2.450±2.413 (2.100, 1.600-2.800) |
| -Missing rate % (n) | 48.508% (8536) | 49.178% (1974) |
| IBIL (umol/L) |  |  |
| -Mean±SD (Median, IQR 25%-75%) | 10.628±5.031 (9.700, 7.500-12.600) | 10.461±4.568 (9.600, 7.500-12.200) |
| -Missing rate % (n) | 48.508% (8536) | 49.178% (1974) |
| TBA (umol/L) |  |  |
| -Mean±SD (Median, IQR 25%-75%) | 4.266±7.991 (2.900, 1.700-4.700) | 4.231±7.363 (2.900, 1.800-4.800) |
| -Missing rate % (n) | 50.742% (8929) | 54.235% (2177) |
| ALT (IU/L) |  |  |
| -Mean±SD (Median, IQR 25%-75%) | 23.869±29.134 (17.000, 12.000-26.000) | 23.216±27.075 (17.000, 13.000-25.000) |
| -Missing rate % (n) | 43.201% (7602) | 42.626% (1711) |
| AST (IU/L) |  |  |
| -Mean±SD (Median, IQR 25%-75%) | 24.008±20.049 (21.000, 17.000-26.000) | 22.520±15.210 (20.000, 17.000-24.000) |
| -Missing rate % (n) | 43.161% (7595) | 42.576% (1709) |
| TP (g/L) |  |  |
| -Mean±SD (Median, IQR 25%-75%) | 70.838±6.904 (71.000, 66.000-76.000) | 71.364±6.122 (72.000, 67.000-76.000) |
| -Missing rate % (n) | 44.888% (7899) | 43.622% (1751) |
| ALB (g/L) |  |  |
| -Mean±SD (Median, IQR 25%-75%) | 41.852±4.852 (42.000, 39.000-45.000) | 42.817±4.493 (43.000, 40.000-46.000) |
| -Missing rate % (n) | 44.832% (7889) | 43.597% (1750) |
| GLU (mmol/L) |  |  |
| -Mean±SD (Median, IQR 25%-75%) | 5.607±1.558 (5.210, 4.800-5.850) | 5.691±1.597 (5.230, 4.800-6.030) |
| -Missing rate % (n) | 49.946% (8789) | 48.356% (1941) |
| UREA (mmol/L) |  |  |
| -Mean±SD (Median, IQR 25%-75%) | 5.280±1.878 (5.000, 4.200-6.100) | 5.400±1.705 (5.200, 4.300-6.200) |
| -Missing rate % (n) | 43.388% (7635) | 42.676% (1713) |
| CREA (umol/L) |  |  |
| -Mean±SD (Median, IQR 25%-75%) | 67.466±28.184 (62.000, 54.000-75.000) | 65.344±21.251 (61.000, 53.000-73.000) |
| -Missing rate % (n) | 43.388% (7635) | 42.676% (1713) |
| UA (umol/L) |  |  |
| -Mean±SD (Median, IQR 25%-75%) | 303.589±93.501 (295.000, 239.000-359.000) | 317.555±88.325 (308.000, 254.000-370.000) |
| -Missing rate % (n) | 43.388% (7635) | 42.676% (1713) |
| K (mmol/L) |  |  |
| -Mean±SD (Median, IQR 25%-75%) | 3.961±0.358 (4.000, 3.700-4.200) | 4.005±0.346 (4.000, 3.800-4.200) |
| -Missing rate % (n) | 43.490% (7653) | 43.049% (1728) |
| NA (mmol/L) |  |  |
| -Mean±SD (Median, IQR 25%-75%) | 140.224±2.348 (140.000, 139.000-142.000) | 141.627±2.471 (142.000, 140.000-143.000) |
| -Missing rate % (n) | 43.490% (7653) | 43.049% (1728) |
| CA (mmol/L) |  |  |
| -Mean±SD (Median, IQR 25%-75%) | 2.358±0.126 (2.360, 2.280-2.440) | 2.408±0.117 (2.420, 2.330-2.490) |
| -Missing rate % (n) | 60.141% (10583) | 68.161% (2736) |
| PP (mmol/L) |  |  |
| -Mean±SD (Median, IQR 25%-75%) | 1.161±0.195 (1.160, 1.030-1.280) | 1.134±0.175 (1.140, 1.010-1.250) |
| -Missing rate % (n) | 60.147% (10584) | 68.161% (2736) |
| MG (mmol/L) |  |  |
| -Mean±SD (Median, IQR 25%-75%) | 0.908±0.084 (0.900, 0.860-0.950) | 0.905±0.069 (0.910, 0.870-0.950) |
| -Missing rate % (n) | 60.152% (10585) | 68.161% (2736) |
| Preoperative LOS (day) |  |  |
| -Mean±SD (Median, IQR 25%-75%) | 2.669±4.093 (2.000, 1.000-3.000) | 1.860±2.518 (1.000, 1.000-2.000) |
| -Missing rate % (n) | 0.000% (0) | 0.000% (0) |
| Gender |  |  |
| -Male % (n) | 38.671% (6805) | 37.693% (1513) |
| -Female % (n) | 61.329% (10792) | 62.307% (2501) |
| -Missing rate % (n) | 0.000% (0) | 0.000% (0) |
| Current smoking status |  |  |
| -False % (n) | 91.794% (16153) | 90.807% (3645) |
| -True % (n) | 1.182% (208) | 1.221% (49) |
| -Missing rate % (n) | 7.024% (1236) | 7.972% (320) |
| Current drinking status |  |  |
| -False % (n) | 92.607% (16296) | 91.405% (3669) |
| -True % (n) | 0.477% (84) | 0.623% (25) |
| -Missing rate % (n) | 6.916% (1217) | 7.972% (320) |
| Marital history |  |  |
| -Unmarried % (n) | 4.450% (783) | 3.139% (126) |
| -Married % (n) | 87.992% (15484) | 87.917% (3529) |
| -Divorced % (n) | 0.216% (38) | 0.399% (16) |
| -Missing rate % (n) | 7.342% (1292) | 8.545% (343) |
| Insurance type |  |  |
| -Non-insurance % (n) | 32.153% (5658) | 28.550% (1146) |
| -Insurance % (n) | 67.841% (11938) | 71.450% (2868) |
| -Missing rate % (n) | 0.006% (1) | 0.000% (0) |
| Department |  |  |
| -Orthopedics % (n) | 12.502% (2200) | 12.058% (484) |
| -Urology % (n) | 27.067% (4763) | 28.401% (1140) |
| -General surgery % (n) | 39.598% (6968) | 35.052% (1407) |
| -Gynecology % (n) | 20.833% (3666) | 24.489% (983) |
| -Missing rate % (n) | 0.000% (0) | 0.000% (0) |
| Anesthesia type |  |  |
| -GA % (n) | 89.464% (15743) | 89.462% (3591) |
| -NB % (n) | 0.727% (128) | 0.100% (4) |
| -LA % (n) | 2.915% (513) | 2.541% (102) |
| -TIVA % (n) | 5.137% (904) | 7.598% (305) |
| -SA % (n) | 1.580% (278) | 0.274% (11) |
| -EA % (n) | 0.176% (31) | 0.025% (1) |
| -Missing rate % (n) | 0.000% (0) | 0.000% (0) |
| Emergency |  |  |
| -False % (n) | 99.017% (17424) | 99.078% (3977) |
| -True % (n) | 0.983% (173) | 0.922% (37) |
| -Missing rate % (n) | 0.000% (0) | 0.000% (0) |
| Operation code (ICD9CM3)^a^ |  |  |
| -(76-84) Operations on the musculoskeletal system % (n) | 8.820% (1552) | 8.221% (330) |
| -(55-59) Operations on the urinary system % (n) | 22.765% (4006) | 24.689% (991) |
| -(40-41) Operations on the hemic and lymphatic system % (n) | 0.813% (143) | 0.374% (15) |
| -(42-54) Operations on the digestive system % (n) | 21.202% (3731) | 19.781% (794) |
| -(60-64) Operations on the male genital organs % (n) | 1.966% (346) | 2.765% (111) |
| -(06-07) Operations on the endocrine system % (n) | 18.759% (3301) | 14.225% (571) |
| -(65-71) Operations on the female genital organs % (n) | 19.697% (3466) | 23.269% (934) |
| -(85-86) Operations on the integumentary system % (n) | 1.216% (214) | 0.772% (31) |
| -(87-99) Miscellaneous diagnostic and therapeutic procedures % (n) | 0.580% (102) | 0.598% (24) |
| -(01-05) Operations on the nervous system % (n) | 3.160% (556) | 3.662% (147) |
| -(00) Procedures and interventions, not elsewhere classified % (n) | 0.017% (3) | 0.000% (0) |
| -(35-39) Operations on the cardiovascular system % (n) | 0.813% (143) | 0.149% (6) |
| -(30-34) Operations on the respiratory system % (n) | 0.085% (15) | 0.050% (2) |
| -(17) Other Miscellaneous Diagnostic And Therapeutic Procedures % (n) | 0.091% (16) | 1.445% (58) |
| -(72-75) Obstetrical procedures % (n) | 0.006% (1) | 0.000% (0) |
| -(21-29) Operations on the nose, mouth and pharynx % (n) | 0.006% (1) | 0.000% (0) |
| -(08-16) Operations on the eye % (n) | 0.006% (1) | 0.000% (0) |
| -Missing rate % (n) | 0.000% (0) | 0.000% (0) |
| Diagnosis code (ICD10)^a^ |  |  |
| -M00–M99 Diseases of the musculoskeletal system and connective tissue % (n) | 4.955% (872) | 5.655% (227) |
| -N00–N99 Diseases of the genitourinary system % (n) | 29.431% (5179) | 32.412% (1301) |
| -C00–D48 Neoplasms % (n) | 23.055% (4057) | 20.179% (810) |
| -K00–K93 Diseases of the digestive system % (n) | 15.520% (2731) | 14.350% (576) |
| -E00–E90 Endocrine, nutritional and metabolic diseases % (n) | 11.871% (2089) | 8.445% (339) |
| -O00–O99 Pregnancy, childbirth and the puerperium % (n) | 2.483% (437) | 3.886% (156) |
| -S00–T98 Injury, poisoning and certain other consequences of external causes % (n) | 4.626% (814) | 3.538% (142) |
| -Z00–Z99 Factors influencing health status and contact with health services % (n) | 2.097% (369) | 3.911% (157) |
| -R00–R99 Symptoms, signs and abnormal clinical and laboratory findings, not elsewhere classified % (n) | 5.154% (907) | 7.050% (283) |
| -L00–L99 Diseases of the skin and subcutaneous tissue % (n) | 0.165% (29) | 0.075% (3) |
| -D50–D89 Diseases of the blood and blood-forming organs and certain disorders involving the immune mechanism % (n) | 0.097% (17) | 0.100% (4) |
| -A00–B99 Certain infectious and parasitic diseases % (n) | 0.051% (9) | 0.050% (2) |
| -I00–I99 Diseases of the circulatory system % (n) | 0.318% (56) | 0.274% (11) |
| -Q00–Q99 Congenital malformations, deformations and chromosomal abnormalities % (n) | 0.091% (16) | 0.025% (1) |
| -J00–J99 Diseases of the respiratory system % (n) | 0.006% (1) | 0.000% (0) |
| -G00–G99 Diseases of the nervous system % (n) | 0.074% (13) | 0.050% (2) |
| -H00–H59 Diseases of the eye and adnexa % (n) | 0.006% (1) | 0.000% (0) |
| -Missing rate % (n) | 0.000% (0) | 0.000% (0) |

^a^The ICD code of operation and diagnosis were retrieved after discharge and not included in the model.

**Table MA1-3. The results of paired-sample T-test for model comparisons**

| **Model** | **Text embedding** | **Mean difference (*P* value)** | | | | | | | |
| --- | --- | --- | --- | --- | --- | --- | --- | --- | --- |
|  |  | **LASSO** | | **Random forest** | | **GBDT** | | **CNN** | **Self-attention** |
|  |  | **With text embedding** | **Without text embedding** | **With text embedding** | **Without text embedding** | **With text embedding** | **Without text embedding** |  |  |
| LASSO | With | / | 0.015 (< .0001) | -0.006 ( .0008) | 0.024 (< .0001) | -0.010 (< .0001) | 0.032 (< .0001) | -0.018 (< .0001) | -0.012 (< .0001) |
|  | Without | -0.015 (< .0001) | / | -0.021 (< .0001) | 0.010 (< .0001) | -0.025 (< .0001) | 0.017 (< .0001) | -0.033 (< .0001) | -0.026 (< .0001) |
| Random forest | With | 0.006 (.0008) | 0.021 (< .0001) | / | 0.031 (< .0001) | -0.004 (.0004) | 0.038 (< .0001) | -0.012 (< .0001) | -0.005 (0.0063) |
|  | Without | -0.024 (< .0001) | -0.010 (< .0001) | -0.031 (< .0001) | / | -0.035 (< .0001) | 0.008 (<.0001) | -0.043 (< .0001) | -0.036 (< .0001) |
| GBDT | With | 0.010 (< .0001) | 0.025 (< .0001) | 0.004 (.0004) | 0.035 (< .0001) | / | 0.042 (< .0001) | -0.008 (< .0001) | -0.001 (0.4673) |
|  | Without | -0.032 (< .0001) | -0.017 (< .0001) | -0.038 (< .0001) | -0.008 (<.0001) | -0.042 (< .0001) | / | -0.050 (< .0001) | -0.044 (< .0001) |
| CNN | / | 0.018 (< .0001) | 0.033 (< .0001) | 0.012 (< .0001) | 0.042 (< .0001) | 0.008 (< .0001) | 0.050 (< .0001) | / | 0.007 (< .0001) |
| Self-attention | / | 0.012 (< .0001) | 0.026 (< .0001) | 0.005 ( .0063) | 0.036 (< .0001) | 0.001 (.4673) | 0.044 (< .0001) | -0.007 (<.0001) | / |
